# Regulation of lung immunity and host defense by the intestinal microbiota

**DOI:** 10.3389/fmicb.2015.01085

**Published:** 2015-10-07

**Authors:** Derrick R. Samuelson, David A. Welsh, Judd E. Shellito

**Affiliations:** Section of Pulmonary/Critical Care and Allergy/Immunology, Department of Medicine, Louisiana State University Health Sciences CenterNew Orleans, LA, USA

**Keywords:** Gut-Lung Axis, intestinal microbiota, immunology, pulmonary infections, pulmonary immunology, dysbiosis

## Abstract

Every year in the United States approximately 200,000 people die from pulmonary infections, such as influenza and pneumonia, or from lung disease that is exacerbated by pulmonary infection. In addition, respiratory diseases such as, asthma, affect 300 million people worldwide. Therefore, understanding the mechanistic basis for host defense against infection and regulation of immune processes involved in asthma are crucial for the development of novel therapeutic strategies. The identification, characterization, and manipulation of immune regulatory networks in the lung represents one of the biggest challenges in treatment of lung associated disease. Recent evidence suggests that the gastrointestinal (GI) microbiota plays a key role in immune adaptation and initiation in the GI tract as well as at other distal mucosal sites, such as the lung. This review explores the current research describing the role of the GI microbiota in the regulation of pulmonary immune responses. Specific focus is given to understanding how intestinal “dysbiosis” affects lung health.

## Introduction

Respiratory tract infectious diseases, such as influenza and pneumonia, result in the death of 3·2 million people annually worldwide (WHO, 2014). Most of the current therapies used in the treatment and management of these diseases are suboptimal as antibiotic resistance, efficacy, and toxicity have been difficult to overcome (Keely et al., 2011). Infection of the respiratory tract represents a breakdown of the host's immune defenses. In addition, non-infections respiratory diseases are the third and fifth (infections respiratory diseases are the fourth) leading causes of death worldwide (WHO, 2014). Understanding the mechanisms that mediate cross-talk between the gastrointestinal (GI) tract and lung defenses and how this interaction facilitates optimal lung health is of growing interest. More specifically, the role of the GI microbiota in mediating, maintaining, and regulating this cross-talk represents an exciting area of research that is poised to aid in the development of novel treatment and management strategies for lung disease.

## The human “super-organism”: The role of the gastrointestinal microbiota in health

The importance of the homeostatic maintenance of human health by the intestinal microbiota has become a topic of great interest (Noverr and Huffnagle, 2004; Lupp et al., 2007; Maslowski et al., 2009; Garrett et al., 2010; Hooper et al., 2012; Bollrath and Powrie, 2013; Sansonetti, 2013). Evolution of an individual's microbiota begins shortly after birth cumulating in a stable adult microbiota by the age of two (Foxx-Orenstein and Chey, 2012). This microbial community includes autochthonous (permanent inhabitants) and allochthonous (transient inhabitants) microorganisms. Microbiota of the human GI tract contains bacterial (microbiota), viral (virome), and fungal (mycobiota) species. Surprisingly, approximately 60% of these organisms cannot be grown in traditional culture (Noverr and Huffnagle, 2004; Hooper et al., 2012). However, new methods known as “microbial culturomics,” which utilize 212 different culture conditions have allowed for a significant advancement in our ability to culture intestinal microorganisms (Lagier et al., 2012). The average human adult intestinal microbiota is composed of approximately 400–1000 species (Noverr and Huffnagle, 2004; McLoughlin and Mills, 2011; Hooper et al., 2012). However, it is estimated that roughly 30-40 species dominate this niche, with bacteria from the genera *Bacteroides, Bifidobacterium, Eubacterium, Fusobacterium, Clostridium, and Lactobacillus* highly represented (McLoughlin and Mills, 2011). In addition, intestinal microbial diversity and composition changes not only along the length of the intestinal tract but is spatially distributed between the mucosa and the lumen of the intestinal tract within each region (Hill et al., 2010; Macpherson and McCoy, 2013). Many environmental factors will drastically alter the normal intestinal microbiota (Noverr and Huffnagle, 2004). Changes in diet, the use of antibiotics, chemotherapy, GI tract infection, and host immune status significantly alter, either transiently or permanently, the intestinal ecosystem (Round and Mazmanian, 2009; Hooper and Macpherson, 2010; Hooper et al., 2012). Alterations of the microbiota that lead to intestinal dysbiosis (a microbial imbalance within the intestinal tract) are characterized by a loss or significant decrease in the amount of beneficial bacterial species and/or an outgrowth or population shift of other species. Intestinal dysbiosis can affect overall health in multiple ways such as growth of opportunistic bacterial pathogens, alterations in host's metabolic profiles, and/or increased inflammation. This review will focus on the microbiota as it affects pulmonary immunity.

## Maintenance of the intestinal microbiota

**Figure 1:**
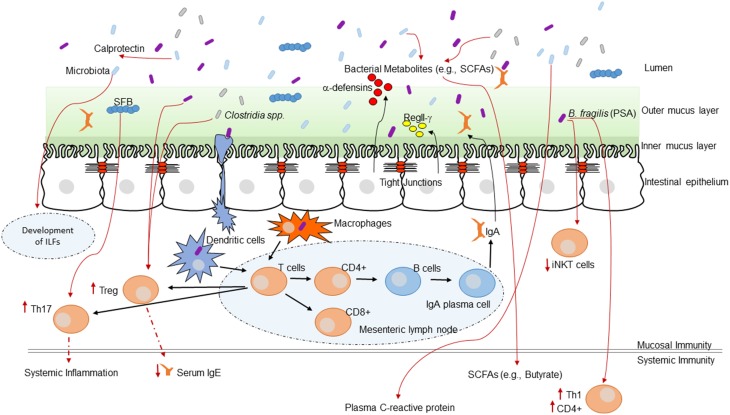
**The intestinal microbiota and the host immune system**. Interaction between the immune system and the intestinal microbiota. Multiple immune effectors function together to minimize bacterial-epithelial invasion. These include the mucus layer, epithelial antibacterial proteins, and IgA secreted by lamina propria plasma cells. Compartmentalization is accomplished by unique anatomic adaptations that limit commensal bacterial exposure to the immune system. Some microbes are sampled by intestinal DCs. The loaded DCs traffic to the mesenteric lymph nodes through the intestinal lymphatic but do not migrate to distal tissues. This compartmentalizes live bacteria and induction of immune responses to the mucosal immune system. Induced B cells and T cell subsets recirculate through the lymphatic and the bloodstream back to mucosal sites, where B cells differentiate into IgA-secreting plasma cells. Thus, the intestinal microbiota shapes host mucosal as well as systemic immunity. ILFs, isolated lymphoid follicles.

Alterations of the intestinal microbiota not only affect the growth of opportunistic pathogens but can have a broad impact on immune status and function within the host (Hooper et al., 2012). The impact of the GI microbiota on host mucosal immunity has been studied extensively in germ-free mice (mice without any intestinal microbiota). Germ-free mice exhibit impaired GI development characterized by smaller Peyer's patches, fewer CD8αβ intraepithelial lymphocytes, underdeveloped isolated lymphoid follicles, and lower levels of the mucosal IgA antibodies (Hooper et al., 2012). The specific microbial molecules or components that inform host immune development are still being discovered and characterized. These interactions are crucial for the maintenance of host-microbial homeostasis. This topic has been reviewed in several recent articles (Round and Mazmanian, 2009; Hooper and Macpherson, 2010; Hooper et al., 2012). Figure 1 highlights a current overview of understanding of how the GI microbiota shape immune responses and how the host immune system shapes the GI microbiota.

## The intestinal microbiota and systemic immunity

Commensal microorganisms modulate host immunity not only in the intestinal tract but at distal sites as well (Kieper et al., 2005). The intestinal microbiota affects systemic immune responses by modulation of several key pathways; expansion of extra-intestinal T cell populations, production of short-chain fatty acids, development of oral tolerance, and control of inflammation.

### Regulation of T cell populations

Expansion and differentiation of extra-intestinal T cell populations are meditated by the intestinal microbiota (Kieper et al., 2005). Several recent studies have shown that the intestinal microbiota is critical for maintenance of T cell subsets that are important for systemic immunity. The intestinal microbiota is required for expansion of CD4+ T cells, regulatory T cells, Th1 or Th2 responses, and Th17 T cells. For example, colonization of germ-free mice with *Bacteroides fragilis* that synthesize PSA results in a higher number of circulating CD4+ T cells and levels of circulating Th1 cells compared to mice colonized with *B. fragilis* unable to produce PSA (Mazmanian et al., 2005). While, colonization of gnotobiotic mice with a cocktail of mouse derived *Clostridial* strains enhances anti-inflammatory signaling by directing the expansion of lamina propria and systemic regulatory T cells (Treg) with an associated increase in IL-10 secretion (Atarashi et al., 2011). The specific Clostridial strain that drives this regulatory affect is not known. Further, mice with high levels of *Bacteroides vulgatus* colonization exhibit a biased T cell differentiation favoring a Th2 over a Th1 phenotype, as characterized by increased levels of IgE, IgG1, IL-4 and decreased IFNγ (Sudo et al., 2002) Finally, colonization of germ-free mice with segmented filamentous bacteria (SFB) induces expansion of the Th17 cell population and a slight increase in Th1 cells (Abraham and Cho, 2009; Wu et al., 2010; Lee et al., 2011).

### Oral tolerance

Development of oral tolerance occurs following oral administration of antigen and represents a local and systemic immunological state of immune unresponsiveness to a subsequent antigen challenge. Low doses of antigen favor active suppression, whereas higher doses favor clonal deletion of antigen-specific T cells. Ingestion of oral antigen induces expansion of Th2 and Th3 T cells and CD4+ CD25+ regulatory cells and latency-associated peptide+ T cells (Faria and Weiner, 2005). Further, individuals with impaired intestinal permeability often have dysfunctional oral tolerance. Impaired intestinal permeability also leads to inadequate production of IgE and recruitment of mast cells in the GI mucosa. Individuals suffering from these conditions exhibit enhanced IgE-CD23-mediated transport across the mucosa and increased levels of inflammatory mediators, such as proteases and cytokines, which further affect intestinal permeability. This leads to an increase in the leakage of allergens and hence contributes to perpetuate the inflammatory reaction (Perrier and Corthésy, 2011). Further, Cassani and colleagues recently observed defective oral tolerance in CCR9-deficient mice (CCR9 targets T cells to the small intestine) and that defective oral tolerance in CCR9-deficieint mice could be restored by transfer of wild-type T cells (Cassani et al., 2011). However, Pabst and co-workers found that CCR9-deficient mice developed normal oral tolerance to ovalbumin. Pabst and co-workers suggest that these differences may be due to the differences in individual strains of CCR9-deficient mice, or that differences in the composition of the microbiota may influence the impact of CCR9 on oral tolerance (Pabst and Mowat, 2012). There are controversial reports on the capacity to induce oral tolerance in germ-free mice devoid of live intestinal bacteria (Walton et al., 2006; Ishikawa et al., 2008). Yet, numerous studies have demonstrated that the intestinal microbiota has a profound effects on the immune system. Therefore, it is conceivable that differences in microbiota composition may also affect oral tolerance.

### Production of short-chain fatty acids

Dietary fermentable fiber content changes the composition of the GI microbiota, in particular by altering the ratio of *Firmicutes* to *Bacteroidetes*. Alteration of the ratio of *Firmicutes* to *Bacteroidetes* directly affect how the gut microbiota metabolize fiber, consequently increasing or decreasing the concentration of circulating short-chain fatty acids (SCFAs). Intestinal microbiota-meditated production of various SCFAs have also been shown to be important for host systemic immunity (Meijer et al., 2010; den Besten et al., 2013; Trompette et al., 2014). More precisely, SCFAs, especially butyrate, seem to exert broad anti-inflammatory activities by affecting immune cell migration, adhesion, cytokine expression, as well as, cellular proliferation, activation, and apoptosis through the activation of signaling pathways (NF-κB) and inhibition of histone deacetylase. In addition, histone deacetylase inhibitors enhance the numbers and function of Treg cells (Meijer et al., 2010). Two recent studies demonstrated that short-chain fatty acids directly regulate/prime the size and function of Treg cell pool in the colon. Both studies showed that mice are protected from colitis through butyrate induced differentiation of Treg cells in a Ffar2-dependent manner (Furusawa et al., 2013; Smith et al., 2013). Furusawa and colleges also demonstrated that treatment of naive T cells under the Treg-cell-polarizing conditions with butyrate enhanced histone H3 acetylation in the promoter and conserved non-coding sequence regions of the Foxp3 locus, which they proposed may be the possible mechanism for how microbial-derived butyrate regulates the differentiation of Treg cells (Walton et al., 2006). Increased levels of butyrate also induce the expression of IL-10, which influence the balance between Th1, cytotoxic CD8+ T cells and Treg cells. Finally, SCFAs are also important in the control of allergic inflammation. Trompette and colleagues found that mice fed a high-fiber diet had increased circulating levels of SCFAs and were protected against allergic inflammation in the lung, whereas a low-fiber diet decreased levels of SCFAs and increased allergic airway disease. Specifically, increased levels of SFAs lead to enhanced generation of dendritic cell precursors and subsequent seeding of the lungs by DCs with high phagocytic capacity, which was accompanied by an impaired ability to promote Th2 cell effector function (Trompette et al., 2014).

### Regulation of systemic inflammation

Several recent studies have provided insight into the role that the commensal microbiota has on influencing systemic inflammation (Noverr et al., 2004, 2005; Ichinohe et al., 2011). Disease severity in animal models of colitis are all dependent on the animal's intestinal microbial communities (Abraham and Cho, 2009; Wu et al., 2010; Lee et al., 2011). For example, germ-free mice with chemically induced colitis exhibit markedly attenuated pathological signs of colitis and restoration of the intestinal microbiota prevents the attenuation. This suggests that the intestinal microbiota is crucial for modulating the host's ability to control inflammation. Further, Verdam et al., found that obese humans exhibit a reduced bacterial diversity, a decreased *Bacteroidetes*/*Firmicutes* ratio, and an increased abundance of potential proinflammatory *Proteobacteria* (Verdam et al., 2013). The shifts in the intestinal microbiota populations were also accompanied by increased levels of fecal calprotectin and plasma C-reactive protein, which suggest that the intestinal microbiota alterations found in obese humans are associated with local and systemic inflammation and that the obesity-related microbiota has a proinflammatory effect (Verdam et al., 2013). Finally, Biagi and co-workers found that by evaluating the correlation between systemic inflammation and the fecal microbiota that about 9% of the variable microbiota was related to the increased levels of pro-inflammatory cytokines IL-6 and IL-8 (Biagi et al., 2010). All of the taxa that showed a slightly positive correlation with either IL-6 or IL-8 belonged to the phylum *Proteobacteria* (Biagi et al., 2010). The intestinal microbiota also has many inflammation-suppressing fractions, which function to; counteract some of the inflammatory bacteria, decrease the inflammatory tone of the system, improve the barrier function of the GI mucosa, and prevent inflammation-inducing components from translocating into the body (Hakansson and Molin, 2011). Furthermore, *Clostridium* cluster XIVa and *Faecalibacterium prausnitzii* has been demonstrated to possess anti-inflammatory effects by inhibiting NF-κB activation and IL-8 secretion and stimulation of peripheral blood mononuclear cells, which ultimately led to an IL-10/IL-12 ratio that favors anti-inflammatory conditions (Sokol et al., 2008). By far, the most studied inflammation-suppressing taxa of the GI microbiota are from the genera of *Lactobacillus* and *Bifidobacterium*. *Lactobacillus* and *Bifidobacterium* will be discussed further in the probiotics section.

While the all of the direct mechanistic contributions of the GI microbiota on systemic immunity beyond the intestinal mucosa remain to be determined, these studies demonstrate that commensal bacteria can impact host immunity beyond the GI tract. Table 1 summarizes our current understanding of the effect of the intestinal microbiota on systemic immunity.

**Table 1:** **Current understanding of the effects of the intestinal microbiota on systemic immunity**.

**Microbial species or product**	**Effects on systemic immunity**
**EFFECT OF THE INTESTINAL MICROBIOTA ON SYSTEMIC IMMUNITY**	
• *Bacteroidesfragitis* PSA+ strains	• Higher levels of circulating CD4+T cells and Thl cells
• *Clostridial* cluster IV	•Enhances anti-inflammatory signaling • Expansion of lamina propria and systemic regulatory T cells • Increases IL-10 secretion
• *Bacteroides vulgatus*	•Influences T cell differentiation favoring a Th2 over a Thl phenotype • Increases levels of IgE, IgGl, IL-4 and decreased IFN-γ
• *Segmented Filamentous Bacteria*	• Induces expansion of the Thl7 cell population • Slight increase in Thl cells
• *Lachnospiraceae*	•Increased production of butyric acid
• Loss of *Akkermansia, Ruminococcus, Pseudobutyrivibrio*	•Correlates with a decreased levels of RANKL • RANKL is expressed by T helper cells and is involved in DC maturation.
• *Parabacteroides distasonis* (Membrane Fractions)	•Decreased levels of proinflammatory cytokines • Stabilization of the intestinal microbial ecology
• *Lactobacillus* spp.	•Promote DC maturation and production of regulatory cytokines • Expansion of CD4+ regulatory T cells
• *Faecalibacterium*	•Inhibition of NF-κB activation through Butyrate production • Interact with DCs promoting induction ofTregs • Induce high amounts of IL-10 in antigen presenting cells • May be able to block expansion of Thl7 cells
• *Bacteroides thetaiotaomicron*	•Can increase gut permeability in mice deplete of microflora. • Increases metabolites involved in gluconeogenesis (succinate)
• *Firmicutes* to *Bacteroidetes* Ratio	•Affects development of Oral Tolerance • Influences how the gut microbiota metabolized fiber • Increased/decreased levels of SCFAs • Increased/decreased levels of fecal calprotectin and plasma C-reactive protein

## Effects of the GI microbiota on pulmonary health

### Priming of intestinal and lung mucosal immunity

**Figure 2:**
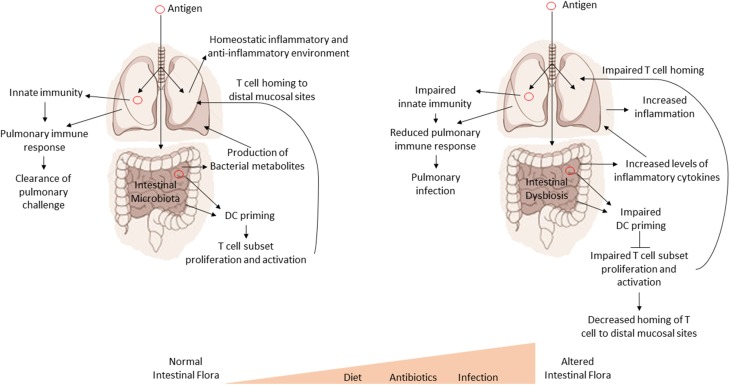
**Intestinal dysbiosis affects systemic immune responses**. A model for the regulatory influence of the gastrointestinal microbiota on systemic immune responses. Antigens are processed by GI tract dendritic cells (DC). The DC then promote the proliferation and expansion of various T cell subsets in response to antigens. T cells then home to sites of infection or antigen exposure. Optimal inflammatory/non-inflammatory conditions and the production of various bacterial metabolites are affected by the composition of the intestinal microbiota. Disruptions in the intestinal microbiota (dysbiosis) lead to impaired proliferation and expansion of T cell subsets, increased inflammation, and loss or imbalance of bacterial metabolites, all of which can have a negative impact on health and systemic immune response.

It is important to understand the cross-talk and collaboration between the GI tract and the respiratory tract at both an immune and microbial level. Numerous studies have shown that fluids, particles, or even microorganisms deposited into the nasal cavity of mice can also be found in the GI tract a short time later (Southam et al., 2002). In fact, as little as 2·5 μl of inoculum into the nasal cavity can later be detected in the GI tract (Southam et al., 2002). Therefore, the GI tract will ultimately be exposed to any pathogen or antigen that is introduced into the respiratory system. This also suggests that the mucosal immune system of the GI tract may serve as a primary sensor of foreign antigens and organisms from the environment. Importantly, disturbances in the intestinal homeostasis by either alterations in the host's genetics or alterations in the microflora could have drastic effects on systemic (e.g., lung) immune responses (see Figure 2). We have also provided a summary our current understanding of the effect of the intestinal microbiota on pulmonary health in Table 2.

**Table 2:** **Current understanding of the effects of the intestinal microbiota on pulmonary immunity**.

**Pulmonary diseases**	**Changes to the intestinal microbiota**
**EFFECT OF THE INTESTINAL MICROBIOTA ON PULMONARY HEALTH**	
• Allergies and Asthma	• Antibiotic-depleted intestinal flora • Significantly moreCD4Tcell-mediated inflammation in responsetoan allergen •Immunological state that is predisposed to toward respiratory allergies
• Infectious Disease	• Antibiotic-depleted intestinal flora • Impaired levels of virus-specific CD4 and CD8Tcell subsets • Treatment with TLR ligands (lung or Intestine) rescued the immune impairment • The normal Gl microbiota is required for expression of pro-IL-lβ and pro-IL-18 • Germ-free mice • Increased susceptibly to pulmonary infection with bacterial pathogens • Increased levels of IL-10 during infection • Suppressed neutrophil recruitment • Increased pathogen growth and dissemination • Treatment with TLR agonist followed restored normal immune responses to bacterial infection • Conventionalized Germ-free mice exhibit normal immune responses to bacterial infection
• Gut-derived sepsis and ARDS	• The “gut-lymph” theory • Macrophages and other immune cells in the intestinal kill the majority of translocating bacteria • Surviving bacteria, cell wall fragments, or protein components may reach the lungs • Increased activation of alveolar macrophages leading to acute lung injury • The “intestinal crosstalk” theory • A three-way partnership amongthe intestinal epithelium, immune tissues, and microflora of the gut • Each factor modifies the others through crosstalk • Normal homeostasis all three components interact normally • Critically ill patients exhibit a loss of the balance between these systems
• COPD	• Cigarette smoke is known to selectively inhibit bacterial growth • Favors a Gram negative bacilli population

### Allergies and asthma

Allergies are often associated with an abnormal Th2 T cell response. Th2 cells are characterized by their ability to produce IL-4, IL-5, IL-9, and IL-13 (McLoughlin and Mills, 2011). The notion that the alterations of immune responses in the gut can directly affect the development of allergic disease in the lung is now widely accepted due to strong epidemiologic (Björkstén et al., 2001) and experimental evidence (Noverr et al., 2004, 2005; Maizels, 2009). A pivotal study reported by Noverr et al. (2004) demonstrated that allergies can develop as a consequence of an altered intestinal microbiota (Noverr et al., 2004). Antibiotic treated mice were given a single oral dose of *Candida albicans*. This significantly altered the composition of the intestinal microbiota (Noverr et al., 2004). Treated animals had more CD4 cell–mediated inflammation in the lung following aerosol introduction of an allergen compared to mice with a normal GI flora (Noverr et al., 2004). This suggests that alterations in the GI flora can facilitate an immunological state that is predisposed to respiratory allergies. There is a growing interest in understanding other T cell subsets in the development of allergy and asthma, specifically the role of Th17 cells and Th9 cells, which may be impacted by GI microbes (Forsythe, 2014). In addition, Vital and colleagues examined the associations between the intestinal microbiota and allergic airway disease in both young and old mice that were sensitized and challenged with house dust mite. They found that the microbial community structure changed with age and allergy development and interestingly that the alterations in the intestinal microbiota from young to old mice resembled the microbial structure of mice after house dust mite challenge. The changes in the intestinal microbial communities were also associated with increased levels of serum IL-17A. Further, old mice developed a greater allergic airway response compared to young mice. Vital and colleagues also suggest the composition of the gut microbiota changes with pulmonary allergy, indicating bidirectional gut-lung communications (Vital et al., 2015).

### Infectious diseases

It is evident that the intestinal microbiota plays a crucial role in the regulation and immune response to respiratory viral infections such as influenza (Ichinohe et al., 2011). A recent study from Ichinohe and co-workers demonstrated that the GI microbiota directly influenced virus-specific CD4 and CD8 T cell subsets in experimentally infected mice (Ichinohe et al., 2011). Treatment of mice with different antibiotic regimens revealed a population of neomycin-sensitive commensal organisms associated with a protective immune response in the lung following influenza infection. Furthermore, injection of TLR ligands, either locally in the lung or at distal sites, rescued the immune impairment in the antibiotic-treated mice. In addition, an intact GI microbiota was required for expression of the proinflammatory chemokines pro–IL-1β and pro–IL-18, which are necessary for influenza clearance (Ichinohe et al., 2010). This suggests that the intestinal microbiota provides microbial signals or determinants that are critical for immune priming and shaping the response to viral pneumonia.

Similar observations regarding the critical role of the intestinal microbiota in the regulation and immune response to respiratory bacterial infections have also been made (Fagundes et al., 2012). Fagundes et al. (2012) employed germ-free mice to analyze the ability of the host to resist bacterial infection. Germ-free mice were highly susceptible to pulmonary infection with the bacterial pathogen *Klebsiella pneumonia*. The enhanced susceptibility to *K. pneumoniae* was associated with increased levels of IL-10, which suppresses neutrophil recruitment, and permits pathogen growth and dissemination. The administration of a TLR agonist followed by LPS inoculation prevented pulmonary *K. pneumoniae* infection, reduced IL-10 secretion, normalized TNF-α and CXCL1 levels, and neutrophil mobilization to the lungs (Fagundes et al., 2012). Germ-free mice that were conventionalized (normal mouse intestinal flora has been restored) had significantly less *K. pneumoniae* in the lungs and blood (Fagundes et al., 2012). Conventionalization also restored neutrophil influx, CXCL-1, TNF-α, and IL-10 to levels found in wild-type mice (Fagundes et al., 2012). These findings suggest that the commensal microbiota maintain host defenses to infectious agents by facilitating a normal inflammatory response to pulmonary pathogens.

### Gut-derived sepsis and acute respiratory distress syndrome

Gut-derived sepsis is the process during which gut-derived proinflammatory microbial and non-microbial factors induce or enhance a systemic inflammatory response syndrome (SIRS), acute respiratory distress syndrome (ARDS), or multiple organ dysfunction syndrome (MODS). Several mechanistic theories of gut-derived sepsis leading to SIRS, ARDS, or MODS, have been postulated (Deitch, 2002, 2012; Senthil et al., 2006; Clark and Coopersmith, 2007; Deitch and Ulloa, 2010). The “gut-lymph” theory proposes that macrophages and other immune cells in the intestinal submucosa or the mesenteric lymph nodes are sufficient to contain the majority of translocating bacteria. However, any surviving bacteria, cell wall fragments, or protein components of the dead bacteria that escape macrophage containment together with cytokines and chemokines produced in the gut, travel along the mesenteric lymphatics to the cisterna chyli. These products then enter into the systemic circulation through the left subclavian vein, via the thoracic duct. Access to the pulmonary circulation leads to uncontrolled activation of alveolar macrophages leading to acute lung injury or ARDS and then MODS (Senthil et al., 2006). Several experimental models support this theory. For example, experimental models of endotoxinemia (Watkins et al., 2008) trauma-hemorrhagic shock (Senthil et al., 2007) or burn injury (Lee et al., 2008) all support this theory. An additional theory put forward by Clark and Coopersmith is the “intestinal crosstalk” theory (Clark and Coopersmith, 2007). This theory assumes a three-way partnership among the intestinal epithelium, immune tissues, and the endogenous microflora of the gut. Within this three dimensional relationship, each factor modifies the others through crosstalk. During normal homeostasis all three components interact normally, which facilitates intestinal crosstalk with extra-intestinal tissues. However, in critically ill patients, loss of the balance between these highly interrelated systems results in the development of systemic manifestations of disease, specifically SIRS, ARDS, or MODS (Clark and Coopersmith, 2007). The mechanisms governing gut-derived sepsis and ARDS are poorly understood and are actively being investigated.

### Chronic obstructive pulmonary disease

COPD is an inflammatory disorder characterized by incomplete reversible airflow obstruction leading to increased mortality and morbidity (Keely et al., 2012; Hui et al., 2013). It has been known for several years that individuals who suffer from COPD have an altered lung microbiome compared to healthy individuals. (Keely et al., 2012; Hui et al., 2013). There is evidence that components of the gastrointestinal microflora, specifically Gram negative bacilli, may also make up a component of the lung microflora and may be increased in individuals with COPD (Keely et al., 2012; Hui et al., 2013). These bacteria are resistant to cigarette smoke and may contribute to severe exacerbations of COPD (Keely et al., 2012; Hui et al., 2013). While no definitive studies on the effect of smoking on the respiratory or intestinal microbiome have been performed, it is possible that smoke-induced changes to the intestinal microbiome may exacerbate COPD symptoms (Keely et al., 2012; Hui et al., 2013).

## Potential mechanisms of GI mediated lung immunity

**Figure 3:**
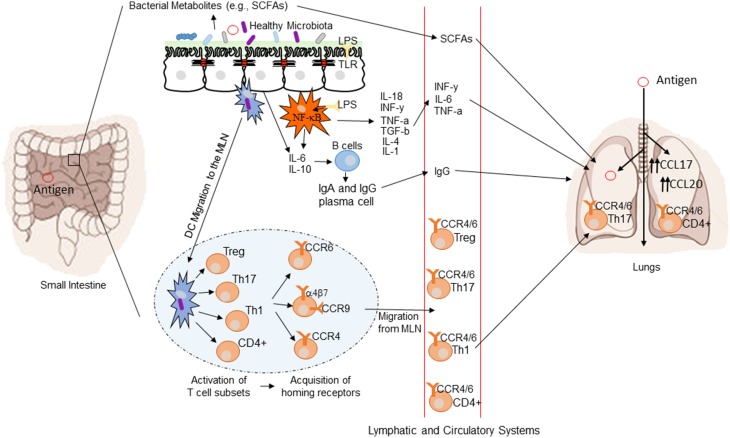
**Conceptual figure of the gut-lung axis**. Proposed model for the regulatory influence of the gastrointestinal microbiota on the immunology of the lung. Microbes in the intestine are sampled by DCs either directly from the lumen or following translocation through M cells to the GALT. A combination of signals from the microbes results in phenotypic changes in the DCs and migration to the draining lymph node. DCs promote the activation of various T cell subsets within the MLN and the production of various regulatory cytokines such as IL-10, TGF-β, INFγ, and IL-6. T cell subsets then acquire immune homing molecules (i.e., CCR9, CCR4, and CCR9). Following immune challenge in the airway, cells activated in the GALT and MLN traffic to the respiratory mucosa via CCR4 or CCR6 where they promote protective and anti-inflammatory responses. In addition, bacterial derived products such as LPS can bind to TLR present on both intestinal epithelial cells and macrophages, leading to the production of various cytokines and chemokines. TLR activation also includes the expression of NF-kB in macrophages. Production of various bacterial metabolites (e.g., SCFAs) also affect the gut-lung axis, as these products are transported to the lung, where they can alter the levels of inflammation.

The mechanisms by which the intestinal microbiota exert a systemic immunomodulatory effect are not fully understood, but several potential pathways may be involved. Highlighted below are several potential mechanisms regulating gut-mediated systemic immunity. Figure 3 provides a conceptual figure of our current understanding to the potential mechanisms involved in the immune regulation along the gut-lung axis.

### Toll like receptor (TLR) activation

The intestinal immune system initiates immune signaling events via the interactions of the gut microbiota with pattern recognition receptors of the innate immune system (i.e., TLRs). TLRs recognize microbial components and trigger inflammatory responses (Abreu, 2010). Different bacterial products, such as lipopolysaccharide, lipoteichoic acid, CpG, peptidoglycan, and polyinosinic:polycytidylic acid, stimulate TLR signaling (Abreu, 2010). One downstream effect of TLR signaling is the activation of the transcription factor NF-κB, which is required for expression of many genes regulating innate immunity and inflammation (Abreu, 2010). The intestinal microbiota is crucial for maintaining normal TLR signaling (Round et al., 2011; Fagundes et al., 2012). Microbiota-mediated activation of antigen-specific CD4 and CD8 T cells, pathogen specific-antibodies, steady state expression of mRNA for pro–IL-1β and pro–IL-18, inflammasome activation, and migration of dendritic cells (DCs) from the tissue to the draining lymph node, which leads to normal T cell priming, all occur in a TLR dependent manner (Ichinohe et al., 2010). Further, intestinal initiated TLR signaling has been shown to induce lung immune responses by Ichinohe and colleagues, who showed that a single dose of LPS delivered intrarectally, restored an immune response in the lung of influenza infected mice (Ichinohe et al., 2010). The elucidation of the major bacterial species or bacterial products necessary to maintain normal microbiota-TLR signaling is an area primed for investigation.

### T and B cell homing

Tissue specific homing of lymphocytes is crucial for an effective immune response and clearance of infection. The localization and homing of lymphocytes is determined by expression of integrin and chemokine receptors, such as CC-chemokine receptor 9 (CCR9) (Christensen et al., 2002; Niess and Reinecker, 2005; Meijerink and Wells, 2010). Specific adhesion and chemokine receptors expressed by lymphocytes allow these immune cells to target tissues that express their cognate ligands and home to areas of high chemokine secretion (Christensen et al., 2002). T cells acquire the capacity to home to non-lymphoid tissues by direct interaction with mucosal DCs at the sites of antigen acquisition. DC-mediated imprinting of T cells confers selectivity for specific non-lymphoid tissues, such as the gut, skin, and lung (Sigmundsdottir and Butcher, 2008; Hart et al., 2010; Mikhak et al., 2013). Dendritic cells sense antigen in tissues before migrating to draining lymph nodes, where they have the ability to activate and influence the differentiation of naïve T cells. Gut DCs promote the expression of α4β7 and CCR9 on T cells, and, in doing so, enable T cells to migrate to the small intestine by homing to the intestinal ligand MAdCAM-1 and chemokine CCL25 (Sigmundsdottir and Butcher, 2008). Alternatively, lung DCs promote the expression of CCR4 on T cells, which allows for activated T cells to traffic into the lung via increased levels of CCL17 (Mikhak et al., 2013).

The ability to induce tissue-specific homing of lymphocytes suggests that mucosal surfaces can not only induce homing back to the site of antigen acquisition but may be able to target distal mucosal sites as well via altered lymphocyte priming. In support of this, Ruane et al. (2013), found that lung DCs can specifically up-regulate the expression of the gut-homing integrin α4β7 on T cells, which guided migration to the GI tract. Consistent with this, intranasal immunization with *Salmonella* induced protective immunity against enteric challenge with *Salmonella* and was dependent on lung DCs (Ruane et al., 2013).

Additional mechanisms allow for lymphocytes to traffic to multiple mucosal sites to combat infection at mucosal surfaces. For example, CCR6 is expressed on immature DCs, most B cells, subsets of CD4+ and CD8+ T cells, and Natural Killer T cells (Ito et al., 2011). The cognate ligand of CCR6, CCL20, is expressed by a variety of epithelial cell types including keratinocytes, pulmonary epithelial cells, and intestinal epithelial cells (Ito et al., 2011). The expression of CCL20 in these tissues remains at a low steady-state level, but is strongly induced by pro-inflammatory signals and TLR agonists originating from bacterial species (Ito et al., 2011). These data suggest that pathogens/antigens encountered in the gut or the lung can prime central and effector memory T cells, which then home to sites of new infection (i.e., sites of inflammation). Thus, CCR6 may be critical for an effective immune response within the gut-pulmonary axis.

## Targeting the intestinal microbiota in the prevention of lung related infectious diseases

### Prevention of respiratory infections with probiotics

The use of probiotics for the treatment of various diseases and for the maintenance of health in general has become an intensely studied area, with broad appeal. Probiotics are now used to treat a variety of aliments including diarrhea, gas, cramping, vaginal yeast infections, urinary tract infections, and to help control inflammatory bowel disease (IBD). Studies are also underway evaluating the benefits of probiotics in the treatment of colon cancer, skin infections, irritable bowel syndrome (IBS), liver disease, rhinoconjunctivitis/rhinosinusitis, and lung health (Forsythe, 2011; Eslamparast et al., 2013; Kumar et al., 2013; Baquerizo Nole et al., 2014; Kramer and Heath, 2014; Sandhu and Paul, 2014). Table 3 describes the current research targeting the intestinal microbiota for the prevention of lung related infectious diseases.

**Table 3:** **The current research targeting the intestinal microbiota for the prevention of lung related infectious diseases**.

**Microbial species or product**	**Effects on pulmonary health**
**CURRENT METHODS FOR TARGETING THE INTESTINAL MICROBIAL FOR TREATMENT OF RESPIRATORY DISEASES**	
• Probiotics (Commonly used probiotics) • *Lactobacillus gasseri* • *Lactobacillus rhamnosus* • *Lactobacillus casei* • *Lactobacillus acidophilus* • *Bifidobacterium lactis* • *Lactobacillus delbruckii subsp. Bulgaricus* • *Streptococcus thermophiles* • *Bifidobacterium bifidum* • *Bifidobacterium breve*	• Enhanced phagocytosis level during normal conditions • Suppressed levels of phagocytosis during allergic conditions • Enhance the production of antigen-specific IgG and IgAantibodies • Suppressed the proliferation of mononuclear cells during inflammation • Reduced burden of pathogens in the lung • Prevents dissemination to the blood • Increased INFγ,IL-6, IL-4,TNF-α and IL-10 levels in BAL • Increased natural killer cell activity
• Oral vaccines against Respiratory Pathogens • *Mycobacterium* spp. • *Francisella tularensis* • *Brucella melitensis* • *Mycobacterium tuberculosis*	• Increased levels of serum antibodies • Increased production of IL-2 and INFγ from splenocytes • Increased levels of antigen-specific antibodies in BAL fluid • Enhanced proliferation of antigen-specific INFγ producing cells • Significant reduction in pathogen burden in the lung following infection
• Probiotic coupled vaccines (Probiotics commonly used as adjuvants) • *Lactococcus lactis* • *Lactobacillus rhamnosus* • *Bifidobacterium animalis* • *Lactobacillus paracasei*	• Increased antigen-specific IgM, IgG (IgGl and lgG2), and IgAantibodies • Serum, BAL, and intestinal lavage fluids. • Stimulation of both Thl and Th2 responses • Increased production of both INFγ and IL-4. • Induces a cross-protective immunity to multiple serotypes • Can be enhanced by a prime-boostvaccination strategy

The benefits of probiotics in the maintenance and regulation of lung health has been described in several studies (Forsythe, 2011, 2014; Yoda et al., 2012; West, 2014). One of the first studies indicating that intestinal microflora may influence lung health came from the study of obese mice. Researchers found that in obese mice the GI microbiota plays an important role in controlling inflammation in the lungs (Yoda et al., 2012). Mice fed heat-killed *Lactobacillus gasseri* had significant increases in pulmonary mRNA expression of cytokines and other immune molecules accompanying the changes in their GI bacterial profiles (Yoda et al., 2012). These results suggest that *Lactobacilli* may stimulate the respiratory immune responses of mice to enhance host defenses against respiratory infection by increasing inflammatory signaling.

Additionally, the probiotic bacteria *Lactobacillus rhamnosus* displays immune stimulatory effects associated with increased resistance to infection (Salva et al., 2010)*. L. rhamnosus* feeding not only attenuated infection with *Salmonella* Typhimurium, an intestinal pathogen, but also conferred resistance to infection with *Streptococcus pneumoniae*, a respiratory pathogen. Probiotic treatment decreased the burden of *S. pneumoniae* in the lung, prevented dissemination to the blood, and increased INFγ, IL-6, IL-4, and IL-10 in bronchoalveolar lavage (BAL) fluid (Salva et al., 2010). Interestingly, while both of the tested *L. rhamnosus* strains improved resistance to intestinal *S*. Typhimurium, only one strain provided a beneficial protective effect against pulmonary infection with *S. pneumoniae* (Salva et al., 2010). This suggests differential probiotic effects and that different infectious diseases may benefit from a specific and unique probiotic treatment regime.

Furthermore, Alvarez et al. (2001) found that mice administrated *Lactobacillus casei* prior to pulmonary challenge with *Pseudomonas aeruginosa* exhibited increased pathogen clearance, phagocytic activity of alveolar macrophages, and IgA in BAL (Alvarez et al., 2001). Similarly, Hori et al. (2001) observed parallel results in murine viral infection models. Feeding mice *L. casei* for 4 months prior to challenge reduced influenza viral titers in nasal washings, which was accompanied by a significant increase in natural killer (NK) activity and IFNγ and TNF-α production (Hori et al., 2001).

### Prevention of respiratory infections with oral vaccines

Over the past two centuries the study and use of vaccines has become increasingly sophisticated. However, even with technological advances many infectious diseases have thwarted the development of effective vaccines. While many routes of vaccination exist, one emerging area of vaccinology is mucosal immunization. This approach provides several benefits over conventional systemic vaccination, including higher levels of antibodies and protection at the mucosal surface. In addition, mucosal vaccines can target specific mucosal surfaces such as the respiratory, genital, or intestinal mucosa. Noteworthy is that vaccination at one mucosal surface often confers resistance at other sites (Ryan et al., 2001; Pasetti et al., 2011). Thus, it is possible to immunize against respiratory pathogens using a gut vaccination strategy.

Several studies have addressed the potential of gut-mediated lung immunity (Doherty et al., 2002; Izadjoo et al., 2004; Aldwell et al., 2006; KuoLee et al., 2007). For example, Doherty et al. (2002) sought to protect the lung against infection with *Mycobacterium tuberculosis* by targeting the gut mucosa. Comparing subcutaneous immunization of mice with oral immunization using a subunit vaccine carrying two *M. tuberculosis* immunodominant proteins, they found that oral vaccination was relatively less effective. However, by using a heterologous priming and boosting strategy, oral immunization induced significant systemic type 1 responses, which were comparable to, or better than, those obtained following standard subcutaneous immunization protocols. Moreover, the immune responses correlated with protection against subsequent aerosol infection with virulent *M. tuberculosis* similar to or greater than that obtained by repeated subcutaneous vaccinations (Doherty et al., 2002).

Evaluating the ability of orally administered live attenuated *Brucella melitensis* to elicit cellular and humoral immune responses and to protect mice against intranasal challenge with virulent *B. melitensis*, Izadjoo and colleges found increases in serum antibodies directed against lipopolysaccharide and non-O-polysaccharide antigens. Additionally, orally delivered *B. melitensis* elicited a systemic response as characterized by increased production of IL-2 and IFNγ from splenocytes. Oral immunization of mice with live attenuated *B. melitensis* protected against disseminated infection and enhanced clearance of the challenge inoculum from the lungs. Optimal protection following inoculation with live bacteria was dose dependent and enhanced by a booster vaccine inoculation. These data suggest that oral immunization may provide protection from pneumonia due to *Brucella* (Izadjoo et al., 2004).

Two additional studies utilizing oral vaccination demonstrated protective immune responses against *Mycobacterium* spp. and *Francisella tularensis* (Aldwell et al., 2006; KuoLee et al., 2007). Both studies showed an induction of antigen-specific antibody responses in the serum and bronchoalveolar lavage fluids, proliferation of antigen-specific IFN-γ producing cells, and an overall reduction in pathogen burden in the lung (Aldwell et al., 2006; KuoLee et al., 2007). These studies demonstrate the potential of GI mucosal vaccination to prevent lung infections. Furthermore, these data suggest that oral vaccination may represent an attractive alternative strategy for the prevention of infection with these two pathogens.

### Prevention of respiratory infections with probiotic coupled vaccines

Preclinical data suggests that vaccine responses may be improved by modulation of the gut microbiota. Several animal studies and human clinical trials have been performed. Mice orally immunized with a recombinant *Lactococcus lactis* modified to express the pneumococcal protective protein A (PppA) in the cell wall were protected from subsequent lung infection with *Streptococcus pneumoniae*. Oral immunization with *L. lactis* expressing PppA increased anti-PppA IgM, IgG, and IgA antibodies in serum, in bronchoalveolar, and in intestinal lavage fluids. A mixture of Th1 and Th2 responses were observed, characterized by the presence of both IgG1 and IgG2a anti-PppA antibodies in serum and BAL and by the production of both IFNγ and IL-4. Furthermore, oral immunization was enhanced by a prime-boost strategy, which induced cross-protective immunity to all *S. pneumoniae* serotypes tested (Villena et al., 2008). Another animal study evaluated a recombinant oral *L. lactis* vaccine against the influenza H5N1 strain. Immunization with *L. lactis* expressing the H5N1 HA antigen induced HA-specific serum IgG and fecal IgA antibody production and mice were completely protected against a lethal challenge of the H5N1 virus (Lei et al., 2010).

Several human clinical trials have been completed in this area. Highlighted below are probiotic-coupled respiratory vaccine human clinical trials. *Lactobacillus rhamnosus* GG pretreatment for 28 days enhanced seroprotection against H3N2 influenza following nasal vaccination with the trivalent live attenuated influenza vaccine containing H1N1-like, H3N2-like, and B-like strains. However, no improvement in protection against the H1N1 and B strains was observed at 28 days and no seroprotection was found at 56 days for any strain (Davidson et al., 2011). An additional study examined the effects of 6 wks. of daily *Bifidobacterium animalis* and *Lactobacillus paracasei* probiotics administration as an immune primer prior to intramuscular influenza vaccination containing H1N1-like, H3N1-like, and B strains. Individuals given probiotic-coupled vaccination had significantly higher vaccine-specific IgG (IgG1 and IgG3), as well as, significantly higher salivary IgA (Rizzardini et al., 2012). However, another study reported no significant difference between the probiotics and placebo groups when evaluating innate immune response to infection (Akatsu et al., 2013). Taken together animal studies and human clinical trial data suggest that coupling vaccines with probiotics may increase the effectiveness of vaccines. However, the data are conflicting. It is clear that optimization of the dosing strategy, as well as, the type of probiotic used will be critical to achieve maximal vaccine effectiveness.

## Conclusions

While we are now beginning to understand the effect of the GI microbiota on lung immunity, characterization of the lung microbiota may also provide valuable insight into lung-mediated immune regulation in response to influenza and pneumonia, as well as, cystic fibrosis, COPD, allergies, and asthma. This review has focused on the mechanisms that govern the effects of the GI microbiota on immune effector and regulatory functions. While there have been significant advancements made in the past 10 years in our understanding of the global impacts of the GI microbiota on human immune function, many questions still remain. Table 4 describes our current understanding of GI regulation of the immune system and areas that remain to be explored.

**Table 4:** **Current understanding of the GI microbiota and future questions**.

**What is known**	**What is unknown**
**THE INTESTINAL MICROBIOTA AND HOST IMMUNITY**	
• The intestinal mucosa is an immune-privileged site	• What is the precise composition of a healthy microbiota required for maximum immune-mediated protection?
• The intestinal microbiota plays an integral part in the development of the immune system • Peyer's patches • CD8αβ intraepithelial lymphocytes • Isolated lymphoid follicles • Mucosal IgA • Vascularization	• How can we to establish and maintain a healthy microbiota throughout life?
• The intestinal flora is required for normal expansion of T cell subsets and induction of anti-inflammatory cytokines • T regulatory cells • Thl7 cells • Thl v. Th2 responses	• What are the direct mechanisms by which gut acquired immunity is translated to protective systemic immunity?
• The Gl microbiota directly participates in protection against allergic, autoimmune, and infectious diseases	• To what extent does the immune system influence other microbiomes [i.e., lung, skin, or urogenital tract)?
• Commensal species of bacteria or bacterial derived products can be used as novel therapeutics forthe treatment of diseases	• Is immune dysfunction a cause or consequence of alterations in the Gl microbiome associated with disease?
• The intestinal microbiota is important for normal systemic immune response	• Where do the microbial signals regulate immune function and immune function regulating microbial composition operate?
• Immune responses generated in the intestinal mucosa are often protective at distal mucosal sites	• How are regulatory cells that develop in the gut directly targeted to distal sites such as, the skin orthe lung?
	• How do we manipulate an unhealthy microbiota to reestablish its positive effects on health?
	• Can we targetthe gut-lung axis forthe treatment of respiratory illness?

## Search strategy and selection criteria

References for this review were identified through searches of PubMed and Google Scholar for articles published from January, 1990, to January, 2015, by use of the terms “Gut-Lung Axis,” “microbiota,” “microbiome,” “oral vaccines,” “oral vaccines + microbiota,” and “microbiota + respiratory infection.” Articles resulting from these searches and relevant references cited in those articles were reviewed. Articles published in English were included.

## Author contributions

DS, DW, and JS conceived of and designed the review. DS and DW did the literature searches. DS, DW, and JS designed and compiled the figures. DS wrote the review.

### Conflict of interest statement

The authors declare that the research was conducted in the absence of any commercial or financial relationships that could be construed as a potential conflict of interest.
